# Understanding Emotion-Related Processes in Classroom Activities Through Functional Measurements

**DOI:** 10.3389/fpsyg.2019.02263

**Published:** 2019-10-23

**Authors:** Victoria Prokofieva, Svetlana Kostromina, Sofia Polevaia, Fabien Fenouillet

**Affiliations:** ^1^Department of Psychology, Laboratory of Human and Artificial Cognitions, University Paris Nanterre, Paris, France; ^2^Department of Psychology, Saint Petersburg State University, Saint Petersburg, Russia; ^3^Department of Neurophysiology, Privolzhsky Research Medical University, Nizhny Novgorod, Russia

**Keywords:** education, neurosciences, emotion-related processes, stress response, assessment classroom activities, heart rate variability

## Abstract

To improve educational research focusing on such complex phenomenon as the interaction of emotion-related processes (affects) and students’ learning classroom activities, the collaboration between educational studies and neurosciences appears particularly relevant. Stress or “stress response” being an emotion-related psychological process (Gross, 2015) and having a neurobiological origin (Selye, 1956) is mostly studied in neurophysiological research using laboratory controlled objective measurements. One of such methods, heart rate variability (HRV) is considered as a reliable neurobiological correlate of stress response as the heart and the brain are directly and indirectly connected, which is advanced by the neurovisceral integration model (Thayer and Lane, 2000, 2009). This article presents an empirical research that uses a neurophysiological HRV method of wireless measurement of stress response in students of 11–12 years old (*N* = 12) during real-life classroom (oral and written) assessment activities and in five different lessons. The stress data were confronted to the analysis of the students’ behavior and the nature of classroom events through a video-based classroom observation. The results indicate that cardiovascular correlates of parasympathetic activity are instantaneous markers of stress response and correspond to real contextual elements of classroom assessment activities, among which the most stressful are *writing a short test*, *an oral reply to the question of the teacher*, *putting up hand to reply*, etc. The stressful factors were highlighted, grouped and ranked. The longest stress duration was registered for *oral reply at the blackboard.* The total stress duration covered 38.8% of time spent in the classroom. This finding suggests that classroom assessment activities are stressful in young students as possibly representing social evaluation.

## Introduction

During the last decades, research in education and cognitive sciences has highlighted the important role that emotions and affective states can play on cognitive processes in learning (Boekaerts, 1993; Pekrun et al., 2002; Woolf et al., 2009; Baker et al., 2010; Calvo and D’Mello, 2011; Schutz and Pekrun, 2011).

Different theoretical approaches emphasize the important influence that affective states play on academic performance. Test anxiety theories have improved the understanding of the predicative role that anxiety plays on performance with its cognitive, behavioral, and physiological components, especially in high-staked (pressure) situations of tests and exams (Wren and Benson, 2004; Cassady, 2009). Consistent with attentional theories (Beilock and Ramirez, 2011), negative thoughts (worries) during a test or exam drain working memory capacity and provoke the attentional biases from a task to intrusive negative thoughts. This reduces performance, particularly on cognitive demanding tasks such as problem solving (Chapell et al., 2005; Hadwin et al., 2005; Beilock et al., 2007; Owens et al., 2008; von der Embse et al., 2015). Cognitive evaluation theory (Linnenbrink and Pintrich, 2002, 2004) postulates that some emotional states can interfere with processing of learning information during its encoding, storage, or retrieval from long-term memory.

More specifically, the field of neurosciences places particular focus on the importance of emotional processes in learning context. “[T]he neurobiological evidence suggests that the aspects of cognition that we recruit most heavily in schools, namely learning attention, memory, decision making and social functioning are both profoundly affected by and subsumed within the processes of emotion” (Immordino-Yang and Damasio, 2007, p. 3). Moreover, research in neurosciences is traditionally interested in understanding the neuronal mechanisms of making decision under stressful (pressure) conditions (Bechara et al., 2000; Starcke and Brand, 2012). These findings stipulate that stress can shift attention from executive functions (governed by the prefrontal cortex) toward sensorial vigilance mode (governed by the amygdala). These processes may impair performance under pressure. Further, neurosciences have also shown a beneficial impact of positive emotions on learning, knowledge transfer and, in a larger sense, on motivational beliefs in education (Fredrickson, 1998, 2001; Goswami, 2005, 2006; Schutz and Pekrun, 2007; Papousek et al., 2010).

Although the research has increased the general understanding of the interactions between cognitive, neuropsychological, and emotional processes in learning, the nature and the influence of affective states and especially short emotional reactions in ordinary classroom activities have not been studied enough and empirical work still remains insufficient (Pekrun et al., 2002; Ainley et al., 2005; Efklides and Petkaki, 2005; Sansone and Thoman, 2005; Linnenbrink, 2006; Fortus, 2014).

Some authors contributed to a better understanding of “academic” emotions (Pekrun et al., 2002), whereas others focused on the emotions that some classroom activities can induce on young students (Fartoukh et al., 2014). Nevertheless, despite the important need to understand classroom emotional processes on the part of educators, teachers, and psychologists, there are scarce studies that are carried out in a real learning environment or classroom setting. “There is limited classroom research available to inform teachers about the range of students’ emotional experiences or how they should respond to their students’ emotional arousal” (King et al., 2015, p. 1886).

Indeed, research in education needs to better understand to what extent the emotional experience of students in classroom activities can impede and disrupt (in case of negative emotional experience) or facilitate and enhance (in case of positive outcome) learning in class. The results of such a research can highlight the following question: “how to integrate affect into existing models of motivation and learning” (Linnenbrink, 2006, p. 307).

However, studying emotional processes in real-life situations (such as class activities of learning and assessment) faces various methodological and experimental difficulties which are mostly due to the complex nature of emotional processes and the multivariable character of school situations (Prokofieva and Velay, 2018). Therefore, it may be very challenging for a researcher to study such an interiorized subjective phenomenon that is difficult to measure and objectivize (Scherer, 2005). There are also methodological difficulties in applying tests used in educational psychology to the real classroom context (Linnenbrink and Pintrich, 2004), where such tests can produce some biases as being not relevant to the school situations or tasks.

Traditionally, in educational research, the perceived emotional states or experiences have been measured by means of self-reported questionnaires. Self-report scales can be either verbal when the participants can verbalize their affective states (Pekrun, 2006) or non-verbal, especially for young children who can use scales with smileys (Burkitt and Barnett, 2006). Self-reports aim at measuring the intensity of emotional states and their components, where the physiological (bodily emotional) component corresponds to the degree of changes in subject’s physical state (“feeling stressed”). Although a self-report method is rather efficient in estimating perceived feelings, the objectivity of such appraisals can be compromised in children and adolescents who may not be fully conscious of physiological processes of their body or, be likely to give socially expected responses. Furthermore, based on retrospective self-judgments (Chafouleas et al., 2009), this method does not intend to measure short changes in affective behavior, or short-lasting emotional stress responses in a class test, or other class activities. Pekrun (2006) underlines that self-reports cannot render real-time estimates of emotional processes and can produce response biases as “emotional processes have limited access to consciousness” (p. 332) and that behavioral and neuropsychological measures are needed to better measure real-life affective processes.

The necessity of physiological markers of emotional reactivity in educational research has been emphasized by the psychological and educational literature (Ringeisen et al., 2018). Most studies focused on laboratory activated variables: challenge or threat (Fonseca et al., 2014), or self-efficacy (Schönfeld et al., 2017), and suggested that these variables are related to neuroendocrine stress responses and affect performance. However, to our knowledge, there are no studies carried out in a real context of in-class activities, which used reliable objective physiological measurement tools. Nevertheless, there is a real need to understand the interaction of objectively measured emotional reactions and classroom activities, especially in young children whose emotional experience can still be “unconscious.”

This article tries to contribute to fill this gap and focuses on studying emotional reactivity (which we will call “stress response”) experienced by students during classroom activities with a special focus on assessment activities. From the emotion-regulation theoretical framework, a “stress response” is defined as a negative or unspecified affective response. Other emotion-related processes (or affects) are emotions (both negative and positive affective responses as sadness or happiness), and moods (longer affective states of both good-bad discrimination) (Gross, 2015). Studying stress response in classroom activities can represent some operational advantages. Contrary to more complex emotional phenomenon such as anxiety, stress response is an instantaneous psycho-physio-neuro-biological reaction to stimulus (classroom event) produced by social evaluation (teacher’s or pair’s judgment). The study of stress can allow a researcher to analyze the following chain of events: classroom activity (event)-stress reaction and to use an objective tool to measure stress manifestations through physiological markers such as heart rate or cortisol level analysis. Moreover, to be taken as a marker of emotional reaction, a physiological correlate must have a psychological trigger and be contextualized (Dickerson and Kemeny, 2004). The methods to be used in educational context of class must imperatively respect these conditions. There are other limitations concerning physiological markers of stress in a real-life context that are important to consider and which will be developed below.

## Materials and Methods

### Physiological Correlates of Stress in a Real-Life Setting

Stress research in youth (children and adolescents) and adults usually considers two main stress regulatory systems: the autonomic nervous system [sympatho-excitatory axis (SAM)] or hypothalamic-pituitary-adrenal (HPA) axis (Rotenberg and McGrath, 2016). The activation of these systems is generally measured though cortisol release tests and heart rate variability (HRV) spectral analysis. Other methods in literature are linked to skin galvanic response (SGR) which registers sympathetic activation or EEG recording the electric cerebral activity of brain regions. To our knowledge, there has still been no evidence in the literature on the possibility of mobile EEG measures in group real-life activity, so this article will discuss mainly cortisol and cardiovascular correlates.

### Cortisol Correlate

Some recent studies in real-life exam settings tried to include objective physiological measurements such as salivary cortisol tests to other psychological self-report variables such as self-efficacy or threat and anxiety appraisal (Ringeisen et al., 2018). Although cortisol correlate contributes to the objective measurement of stress reactions, this method is rather limited in studying classroom emotions as it does not allow measuring short-term changes of behavior during an exam or a test, or during a short class assessment activity.

In psychophysiological research, cortisol analysis is often used in the laboratory studies where the mental stressor is reconstructed artificially (Campbell and Ehlert, 2012). This method measures long-term emotional states like exam-related anxiety before and after the event, as it assesses the hypothalamic-pituitary-adrenal (HPA) axis activity (the second stress stage of Selye, 1956). Thus, the meta-analysis of the studies on the relation between an acute stressor and a cortisol release attests that the interval between cortisol assessments usually varies from 30 min to a few days, with the average cortisol assessment occurring 29.9 min (SD 16.2) from stressor onset (Dickerson and Kemeny, 2004). Cortisol release into the bloodstream is the sign of HPA adrenal axis activation. It is triggered by the corticotropin releasing hormone (CRH), stimulating the anterior pituitary to secrete adrenocorticotropin hormone (ACTH) (Lovallo and Thomas, 2000; Sapolsky et al., 2000). It is, therefore, questionable to be taken as a measurement tool of a stress reaction at the moment of the introduction of the stressor (referred mostly to the SAMs, sympathetic-adrenal-medullary axes). It will not enable the registration of the rapid bodily emotional changes during a very short class test (within 20 min from stressor onset) or a short oral class assessment.

Other physiological method used to measure emotional reactivity or stress is heart rate variability (HRV) analysis.

### Heart Rate Variability as a Marker of Stress Response

According to appraisal theories (Lazarus, 1993), a stress response is a reaction of the subject to the circumstance that he/she appraises to exceed his/her capacity to cope (Lazarus, 1993; Gross, 2015).

In neurophysiology, acute stress corresponds to the activation of the autonomic nervous system and can be measured by heart rate variability (HRV), an indicator of a cardio-autonomic control of the adaptive processes (Pumprla et al., 2002).

Mental acute stress and changes in cardiovascular parameters are closely related, as HRV parameters are considered as a rather sensitive and selective measure of short stress-related periods of stress reactivity (Hjortskov et al., 2004; Taelman et al., 2011). Heart rate variability (HRV) refers to alterations in heart beat time intervals and provides quantitative markers of autonomic regulation (Akselrod et al., 1981). This physiological correlate has been suggested to be an appropriate index of the degree to which this system provides flexible, adaptive regulation (Thayer et al., 2012) as reflecting the changes in sympathetic and parasympathetic activity which characterize the autonomic reactivity. HRV is mostly linked to two main axes of stress (Selye, 1956), i.e., the fight-or-flight response [or activation of the sympathetic adrenal medullary (SNS) and the hypothalamic-pituitary-adrenal (HPA) axis] (Marques et al., 2010).

Recent meta-analysis literature on the associations between acute mental stress and short-term heart rate variability (HRV) measures in time, frequency, and non-linear domain confirms that instantaneous changes in heart rate regulations is a response to the introduction of a stressor. This can be a reaction to physical stress (pain) (Koenig et al., 2014) or mental load (Castaldo et al., 2015; Kim et al., 2018). The time and frequency domain analysis is used to study the disorganization of cardio-vascular activity. Such situations are characterized by HR (heart rate) increase and depression of HRV fluctuations at high frequencies during stress. Parasympathetic balance ratio [low frequency (LF)/high frequency (HF)] is generally proved to increase under acute mental stress, suggesting a sympathetic activation and a parasympathetic withdrawal (Berntson et al., 2016).

However, it is very important to note that there are some critical discussions in the recent literature concerning the interpretation of HRV variables (Heathers, 2014; Laborde et al., 2017). This questioning is based on the nature of sympatho-vagal balance (LF/HF ratio) (Billman, 2013; de Geus et al., 2014) and the physiological underpinning of LF (de Geus et al., 2014), thus representing a “loose relationship” between LF power and sympathetic nerve activation (Billman, 2013). Some authors suggest taking into consideration other HRV variables to support the analysis. Thus, Laborde et al. (2017) strongly recommend to a researcher “to adopt HRV indices that reflect clearly identified physiological systems with a theoretical underpinning such as the indices of vagal tone (i.e., RMSSD, peak valley, and HF-HRV)” (p. 5).

Given all these arguments, the authors of the present study suggest to account for two HRV variables as a marker of the activation of the adaptation processes and, therefore, a stress response would be defined as the simultaneous increase of LF/HF (which would reflect the activation of sympathetic and withdrawal of the parasympathetic systems) and the decrease of TP (total power) which reflects the inhibitory influence of endogenous opioids system on the activation of the hypothalamic-pituitary-adrenal (HPA) axis of stress (Drolet et al., 2001; Bodnar, 2013, 2016; Valentino and Van Bockstaele, 2014). These patterns as a marker of stress response were experimentally observed and validated in different real-life stressful situations (bus drivers facing unexpected situations on the road, students presenting in front of the class, etc) (Polevaia, et al., 2017).

If these HRV variables are indeed a marker of situational acute stress, it would suggest the possibility of using this method in situations of exam-related stress and in other stressful situations in real-life classroom activities.

The aim of this study is twofold. At first, it proposes to investigate on the relevance and effectiveness of wireless HRV measurement as being a marker of stress responses in children in the context of a real classroom activity. The main question is raised in relation to this: whether the HRV method used in this study (Polevaia, et al., 2016, 2017) enables to register instantaneous changes in emotional reactions in students during classroom activity.

The second aim lies within the field of education, and studies what contextual elements of classroom activity correspond to the registered periods of stress, what these activities are and if they can be identified, categorized, and ranked. The study is purely exploratory and emphasizes the necessity of the interplay between experimental neurophysiological research (quantitative methods) and the methodology used in educational study (qualitative methods).

### Participants and Procedure

The study took place at a public secondary school (called Gymnasium) of the City of Saint Petersburg (Russian Federation) which has approximately 800 students aged between 11 and 18 years old. The students came from diverse socio-economic backgrounds.

Three classes participated in this study (*N* = 96) aged 11–12 years old (*M* = 11.7). The students were in the Fall semester of Year 5 (which corresponds to the first year of compulsory secondary school system in Russia).

This Gymnasium has a special contract agreement with the Department of Psychology of St Petersburg State University. This facilitated the implementation of this study, as the head and the staff of the school are in contact with the university and the students are used to the presence of the psychologist in the class. Students of this school and their parents are systematically asked to sign the written consent forms which are valid for the whole year. More specifically, written informed parental consent was obtained for all research activities in relation to this study. The study was approved by the Ethics Committee of the University of St Petersburg.

The administration of the Gymnasium was notified on the purpose of the study. The pedagogical staff of the classes participated in the study were also notified, and they showed a great interest. They were promised by the experimental team to receive a feedback presentation of the results at the end of the experiment.

On the day of the study, the students of three classes were told that the team from the university would join their lessons in order to observe their classroom activities. The time table was not modified that day, and the lessons were chosen based on their usual hours.

The experimentation team visited five lessons: three Russian lessons (native language for all students) in three different classes, a Mathematics lesson, and a foreign language lesson (English). The duration of a normal class session is 45 min in this school. Four members of the experimental group sat on the class at the back. They observed the activities and students’ behavior and made sure the EEG recording devices worked properly.

The total number of students whose EEG was registered throughout the day was 12 (six boys and six girls). A randomized method was used in selecting the participants. The 12 selected students had an average mark corresponding to 4/5 in the three subjects and represented the middle range of school performance (68% of students) As the girls in these classes had a slightly higher performance level than the boys (4.38 and 4.04, respectively, *t*(74) = 2.70, *p* < 0.01), we chose to select two boys and two girls within the middle-range performance to level out gender differences.

Before the beginning of the lessons, four students from each of the three groups (two girls and two boys) were taken to another room and were informed by the team that the school “was about to buy new equipment for measuring heart rhythms and that their help in checking how equipment worked would be precious.” The students were curious and accepted with pleasure, and the EEG wireless sensors were put on. The girls and the boys were equipped separately to respect sex differences. From the moment of being connected, the sensors started on-line registration of the students’ cardio parameters before the students entered the classroom. The four students joined the rest of the class and the lesson started. The rest of the students of the class were not informed about their classmates having some registration tools and had no possibility to converse with them. All students of the class were doing the same classwork and under the same conditions. No special instructions from the research team were given to the teachers. The lessons were carried out according to the learning activities planned by the teacher for that day. For the video observation, a static video camera was placed in the corner of the classroom discretely before the beginning of every session.

#### Data Collection

1. Wireless telemetric ECG registration with further spectral analysis of HRV
2. Video registration by a static camera
3. Direct observation in class

#### Variables Measured

Two different types of dependent variables were calculated: (1) stress responses extracted from the HRV analysis (in terms of the number of stress events and stress duration) and (2) behavioral variables corresponding to the events occurred during each lesson (mostly learning and assessment activities).

#### Stress Responses

Stress manifestations were recorded in 12 students [four students per each class group (*n* = 25)].

The telemetric electrocardiogram (EEG) data were registered by means of a small sensor ZephyrBioHarness clipped on to an elasticized chest strap which was fitted around the participant’s thorax. The data were transferred to an Android mobile device *via* a wireless protocol Bluetooth SPP 2.4 GHz and then to a central server. The data registration, storage, and following transfer from the mobile device to the central server were provided by the software HR-Reader (Parin, et al., 2014; Polevaia, et al., 2016, 2017). The presence of stress response periods was measured by the heart rate variability (HRV) spectral analysis. From heart rate recordings, HRV was extracted, using inter-beat interval data. The received RR signal was cut by a time window for 100 s with a time shift of 10 s.

Frequency-domain measures of HRV were based on power spectral analysis (ms^2^) derived using the Fast Fourier transformation. The following indices of heart rate variability were recorded:

1. spectrum power in very low frequency (VLF; 0.003–0.04), in low frequency (LF; 0.04–0.15 Hz), and in high frequency (HF; 0.15–0.40 Hz) regions,
2. total power of the spectrum (TP = VLF + LF + HF),
3. parasympathetic balance or the LF/HF ratio (relation of the spectrum powers in low-frequency and high-frequency regions LF_HF = LF/HF). All frequency measures were normally distributed.

Taking into account the specificity of the cardiac signal obtained in real-life contexts (namely, the presence of a non-stationary property and a large number of transition regions), a set of specialized spectral methods for signal processing is suggested:

1. Continuous wavelet transform (CW method or Morlet wavelet) for analysis of amplitude modulations of RR intervals and spectral components of rhythmograms.
2. Dynamic spectral analysis, which synthesizes Fast Fourier transformation algorithms and Lomb-Scargle periodograms, for analysis of rapid changes in the structure of HR. In our case, dynamic spectral analysis was performed in a specialized program incorporated in the LabVIEW environment.

As a result, the temporal dynamics of the power characteristics of the vibration spectra of the RR intervals were analyzed, namely: the total power of the HRV spectrum (TP, ms^2^); LF, ms^2^; HF, ms^2^; and the ratio of the power of the rhythmogram spectrum.

The baseline was recorded based on rhythmogram within periods of passive activity (without any significant events taking place). The selection of these fragments was based on:

1. duration (over 5 min).
2. acceptable distortion of RR intervals: less than 5%.
3. student being in a calm position: sitting, and passively listening to the teacher or to his class mates.
4. the HRV parameters of the beginning of the stress-response were compared to HRV of passive listening.

The beginning of the periods of stress response corresponded to a simultaneous increase of LF/HF suggesting the sympathetic activation and the parasympathetic withdrawal (Berntson et al., 2016) values with the fall in the total power spectrum of HRV (TP). This dynamic pattern of RR intervals as being characteristic for stress was evidenced by the previous studies (Polevaia, et al., 2016, 2017) and determined as common not only in laboratory studies but also in a series of stressful and extreme situations in a real on-line monitoring in bus drivers (at the moment of a sudden maneuver) in firemen during training in the gas-smoke chamber and in students during public oral presentations (Bakhchina, et al., 2014a,b; Polevaia, et al., 2016).

It is also worth-noticing that the recording took place in a natural classroom setting during a whole academic day. The class activity of every lesson lasted 45 min, which enables observation of the behavior of a student in all situations: sitting passively, moving, replying, writing, etc.

### Data Analysis

#### Stress Data

**Figure 1:**
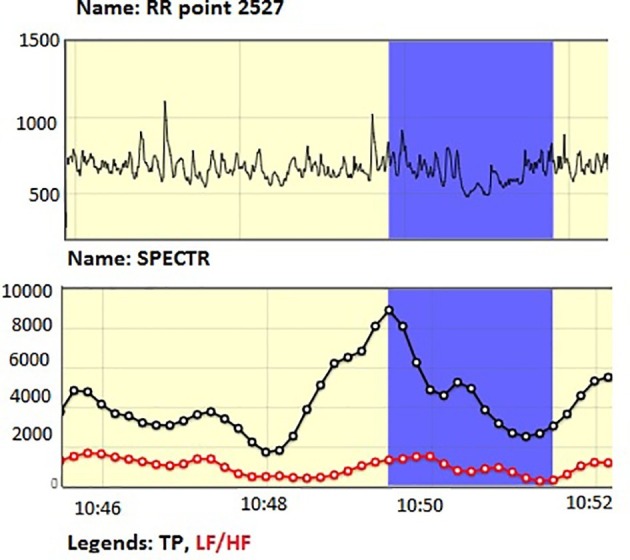
Graphics showing a stress response pattern of a student writing the class test. Beginning of the stress at approximately 10:47:48, ending at 10:49:48. Total duration 2 min (120 s).

Initially, the number of students whose EEG was registered was 12 (six boys and six girls). However, one female student’s stress data were incomplete (interrupted recording and only a partial data available), so the EEG registrations of only 11 students were analyzed. See below an example of a graphical illustration of a stress pattern (Figure 1) with its standardized HRV spectral variables (Table 1).

**Table 1:** Standardized HRV spectral variables for a stress period in a student writing the test.

Time	LF	HF	TP	LF/HF
10:47:48	3590.84	802.07	8948.87	4.48
10:47:58	2875.67	609.33	8126.88	4.72
10:48:08	2030.49	401.26	6287.22	5.06
10:48:18	1551.73	303.08	4897.32	5.12
10:48:28	1358.26	353.05	4616.36	3.85
10:48:38	1315.45	483.06	5281.49	2.72
10:48:48	1456.27	573.02	4967.60	2.54
10:48:58	1699.34	569.96	3883.13	2.98
10:49:08	1783.42	550.04	3189.84	3.24
10:49:18	1586.30	650.28	2706.43	2.44
10:49:28	1194.27	842.04	2532.22	1.42
10:49:38	880.38	934.22	2681.17	0.94
10:49:48	912.10	843.64	3065.07	1.08

#### Behavioral Data

For the behavioral data, classroom activity was observed and analyzed using the time-stamped videos of each lesson. The principal classroom activities were defined by the principal researcher and grouped into seven main categories (class events), with a special focus of assessment classroom activities.

The following codes of classroom assessment activities were defined based on their concurrency. All assessment activities were considered:

Code 1: Writing a short class written test.

The students were asked to do a written work on text (finding the information or highlighting a studied grammar point in the text) or fill in a short quiz. This activity usually lasted 5–10 min with a group checking and correcting at the end.

Code 2: Responding to the teacher’s question.

This corresponded to the teacher’s asking subject-related questions on and asking students to answer in front of the class.

Code 3: Interacting with the teacher.

Any type of teacher-students’ interactions: teachers comments on the class activity or that a particular student, criticism, encouragements, etc.

Code 4: Interacting with another student (pair work).

Mostly pair assessment activities concerning class work (dialogues in English, pair work on text and questions asked by a teacher, etc.)

Code 5: Putting up hand to answer question.

To respond to the teacher’s question, some students wanted to reply or to comment on the subject in front of the class.

Code 6: Answering at the blackboard.

The pupil was asked to go to the blackboard and to write the answer or to draw a figure on the board.

Code 7: Another student at the blackboard.

While a class mate is at the backboard, other students follow by working at the same time or by watching the student doing the exercise. Some students tried to help or to prompt a right answer to the student in question.

Two external expert judges were asked to view the time-stamped videos of the five filmed lessons (total duration of registration: 225 min.) and to use the above predefined codes to identify every event that took place during each lesson with a special focus on the behavior of the students wearing sensors. Thus, for example, when the student wearing a sensor put up his (her) hand to reply, the judge noted the code of the event (C5), its beginning, and its end (corresponding to the moment when the student shifted to another activity) using the time indicator on the video recording. Judge 1 thus identified 116 behavioral events corresponding to those of the students with sensors [total duration: 10,880 s (181.33 min)], whereas Judge 2 identified 98 [total duration: 10,443 s (174.05 min)]. These data were analyzed crossing stamped video registrations, judges’ tables of events, and the HRV rhythmograms (indicating the time of students’ stress response and its duration). This enabled to see if there was a correspondence between observed behavior and recorded stress response (HRV).

#### Inter-Rater Agreement Between the Judges

We found some difficulties to match the events identified by both judges given that the events identified by both judges were not strictly identical in terms of duration. A judge could distinguish two events where another could identify and code it as one event for the same period. However, we analyzed if there was an inter-rater agreement between the judges for the events with at least partial concordance (temporal overlap in time) based on the seven coding categories pre-established before.

The Cohen’s kappa was calculated to measure the degree of agreement. The Cohen’s kappa was 0.71 (*p* < 0.001), which illustrates a good degree of agreement between the judges.

## Results

### Stress Response

The analysis of the stress data showed 37 stress events in the students wearing sensors. Total stress duration was 4,827 s (80.45 min) out of 13,500 s (225 min) of total lessons’ duration. It represented 38.76% of time spent in classroom activity (See Table 2).

**Table 2:** Number and stress duration by lesson (total and mean).

Lesson	Number of stress events	Minimum duration (s)	Maximum duration (s)	Total duration (s)	Mean duration	Standard deviation
English	7	8	295	908	129.71	90.49
Maths	1	64	64	64	64	—
Russian 1	7	55	300	1,191	170.14	81.58
Russian 2	13	58	240	1,391	107	54.71
Russian 3	9	57	314	1,273	141.44	87.43
Total	37			4,827		

#### Total Stress Duration According to the Nature of the Lesson

The most stressful were three lessons of the native language with a total duration of 1,391 s (23.18 min), 1,273 s (21.22 min), and 1,191 s (19.85 min), respectively. The total duration of stress for all Russian classes was 1,285 s (21.42 min). The least stressful class was the Mathematics class with total duration of 64 s (1.06 min). The Russian classes were also on average more stressful than the other classes [mean of 132.93 s (2.21 min) and 77.66 standard deviation], compared to 129.71 s (2.16 min) for English and particularly 60 s for Mathematics.

### Behavior Results

#### Concordance Between Stress and Behavior

One of our main questions in this research was whether there could be a match between observed behavior and stress. To answer this question, we assessed whether there was a temporal overlap between the behavioral data assessed by both judges and the stress data (HRV).

There is concordance when a behavioral event coincides in time with a stress event. As far as the stress event can start simultaneously or shortly after a behavioral event begins (as it caused by the latter), a gap of 10 s is considered acceptable between the beginning of the two events (stress and behavioral). It is also coherent with the stress events measurement as the HRV spectral analysis provides data in 10-s windows.

J.1 identified 30 of the 37 stress events (i.e., 81.1%).

J.2 identified 36 of 37 stress events (i.e., 97.3%).

For both experts, within 29 out of 37 stress events, there was a clear concordance with a behavioral event. The concordance analysis will thus cover these 29 events (E29).

The Cohen’s kappa was calculated to measure the degree of agreement for these 29 events. The degree of agreement, *k* = 0.79 (*p* < 0.001) is even better in E29 (which coincides with periods of stress) than for the full range of events (37). There seems to be no ambiguity on the nature of these events.

The events 1, 2, and 5 account for more than 70% of all events, reported by both the judges (See Table 3). It means that the activities: writing short test and oral questioning of the students in front of the class were the most frequent during all lessons. The category 5 (putting up hand to respond to the question) can be also seen as referring to the oral questioning.

**Table 3:** Code events E29 (J1 and J2).

Code event	Number of events (J1)	Percent	Number of events (J2)	Percent
1	7	24.1	7	24.1
2	5	17.2	6	20.7
3	2	6.9	3	10.3
4	3	10.3	3	10.3
5	7	24.1	7	24.1
6	1	3.4	1	3.4
7	4	13.8	2	6.9
Total	29	100	29	100

As it can be seen in Tables 4, 5, the largest number of C2 (giving oral reply) and C5 (wanting to answer) as well as all events C1 (writing shot test) mostly occurred during the three Russian classes [C1-11/11(J1) and 12/12(J2)]; C2[4/5(J1) and 5/7(J2)]; C5[4/7(J1) and 3/4(J2)]. This can possibly explain why Russian lessons were the most stressful both in stress occurrence and in stress duration. The mean duration of stress period in the English lesson was also rather significant, but the most frequent event was different C4 [interacting with another student (pair work): 3/7 (J1) and 2/6 (J2)].

**Table 4:** Number of classroom events (C1-C7) according to the lesson (J1).

Lesson/code	1	2	3	4	5	6	7	Total
English	0	0	1	3	2	0	1	7
Maths	0	1	0	0	0	0	0	1
Russian 1	4	0	0	0	2	0	0	6
Russian 2	6	1	2	1	1	0	2	13
Russian 3	1	3	0	0	2	2	1	9
Total	11	5	3	4	7	2	4	36

**Table 5:** Number of classroom events (C1-C7) according to the lesson (J2).

Lesson/code	1	2	3	4	5	6	7	Total
English	0	1	1	2	1	0	1	6
Maths	0	1	0	0	0	0	0	1
Russian 1	5	1	0	0	1	0	0	7
Russian 2	6	1	3	0	1	0	2	13
Russian 3	1	3	1	0	1	2	0	8
Total	12	7	5	2	4	2	3	35

## Discussion

This study aimed to contribute to the research in emotions in education. It particularly focused on the interaction of situational bodily emotional reactions such as stress responses and school classroom activities. Educational research needs to find objective methodologies to measure emotional processes which, being of psychological origin also have neurobiological manifestations (Dickerson et al., 2004; Prokofieva and Velay, 2018).

We tested in our study a HRV telemetric methodology of measuring stress response stemming from neurobiological research. As a result, this study can raise interest for neurosciences in terms of operationalization of laboratory neurophysiological methods in the context of real-life activity and social interaction, i.e., a classroom.

We used a wireless telemetric method of the electrocardiogram (EEG) registration which is less invasive than other static devices as it meant for the students that only a small sensor was clipped on to an elasticized chest strap around their thorax. As the data were transferred to an Android mobile device *via* a wireless protocol Bluetooth at a distance of 10 m, the students were free in their movements and did not pay attention to the registrations while classroom activities.

We did not use any self-estimate scales in this study as we did not want the students to be aware that the study was designed to observe stress in class and that their stress data were being recorded. Doing so enabled us to erase a possible bias that self-estimate methodology is known to produce (Pekrun, 2006). Even if such data could have enriched the study of possible correlations between physiological and perceived stress, our choice of not to use self-estimates was based on the inconsistency of the results of previous studies. They conclude that the association between perceived stress and both SAM and HRA axis activity in children and adolescents is unclear (Rotenberg and McGrath, 2016). Anyway, further research can focus on this inter-relation and test these hypotheses.

We, nevertheless, respected the conditions necessary for a physiological correlate used in this study to be considered as a marker of emotional reaction (Dickerson and Kemeny, 2004). It had a psychological trigger (assessment events being situations of social evaluation) and was contextualized (correlated with real-life classroom activities).

The results of the stress response analysis of 11 students and its concordance with classroom events suggest that the method of spectral analysis of HRV measures efficiently the activation of adaptive processes in real-life setting. The analyzed HRV variables and the stress pattern which was experimentally identified by the previous studies (Polevaia, et al., 2017) appeared the same in young students. Taken into consideration the recent discussions on stress markers in the neurophysiological literature (Laborde et al., 2017), such methodology can give interesting direction for further studies in educational contexts.

However, the primary purpose of this study was to try to answer an educational question: Are classroom assessment activities stressful? The HRV method made it possible to measure both stress occurrences and the duration of each stress period. Thus, 37 stress events in the students wearing sensors were registered. Total stress duration in all students was 4,827 s (80.5 min) of total lessons time (13,500 s, 225 min). It represents 38.8% of time spent in classroom. This finding challenges the previous educational literature that states that classroom tests, essays, and evaluations are not high-stakes and are thus not very stressful (Hodge et al., 1997; McDonald, 2001). Moreover, these events appeared to be rather stressful as they may be threat-or shame-inducing as they represent for students the situations of social evaluation (by teacher, pairs, or others). Consistent with the social evaluative and social self-preservation theory, such emotions are known to result in “distinctive patterns of activation in peripheral neural systems such as the autonomic nervous system” (Dickerson et al., 2004, p. 1193).

Can stressful class assessment situations be classified and is it possible to hierarchize them?

The good concordance between the majority of behavioral and stress events enabled us identify 29 (of 37) class events and their perfect concordance with stress events and on which our judges had fully agreed.

Our findings showed in terms of frequency of classroom activities that “Writing a short class test” accounted for 24.1% of all events, “Responding orally to the teacher’s question” for 17.2 (J1) and 20.7% (J2), respectively and “Putting up hand to answer question” for 24.1%. Together these three assessment activities represent 70% of all stress-related events.

Interestingly, in terms of total stress duration, the lessons of the native language (Russian) appeared to be the most stressful (mean stress duration of 132.93 s). It can be explained by the nature of the most frequent activities taking place during these lessons, i.e., a written test, which all occurred in Russian classes [code C1-11/11(J1) and 12/12(J2)].

Further, the stress duration during English classes was also significant (908 s), but the event that provoked these stress responses was different: Code 4 (pair work interaction). The lesson of Mathematics was the least stressful (64 s) explained by an almost complete absence of assessment activities during this class. Finally, the most stressful of all assessment events in this study was the “Response at the blackboard” which occurred twice during a class of Russian (stress duration: 123 and 314 s, respectively).

Although this study helped to observe some interesting phenomenon in relation to emotional processes in students, we would nevertheless be careful enough not to generalize these findings, as stress behavior can vary in individuals and depends on other factors not controlled in this study (e.g., health, recent stress-related experience or chronic stress, fatigue, etc.). More stress data and a larger sample would provide better possibilities for statistical analysis. However, in this study, the focus was to explore a stress-measurement method and may contribute in designing further study on a larger scale.

We are also aware that the presence of researchers in the class could have an impact on pupils’ behavior. At the same time, the HRV registration which lasted all school day proved the contrary. If some pupils had been stressed by the presence of the researcher, the changing of stress response parameters would have been recorded. For example, the students would have been more stressed in the beginning and less stressed in the end of the day or vice a versa. However, no increase or decrease of stress response dynamic was registered. It suggests no objectively registered impact of researchers’ presence.

As this study is exploratory, it could not focus on other hypotheses and account for more variables. Further research could shift from semi-experimental protocol to an experimental one with controls for all variables. For example, it would be interesting to design the same pedagogical scenario for different subjects (with the same number of assessment events) for a better comparison of stress responses on the same activities in different classes. Another possibility, as mentioned before, would be to check the hypothesis of the correlation between perceived stress and objectively measured stress in young students. Furthermore, measuring physiological bodily emotional manifestations could introduce an additional objective variable to the research on test anxiety and to contribute to the “daunting task” (Koster, 2012) to select specific and most appropriate measures of such multifaceted concepts as anxiety and stress.

From the neurophysiological point of view, it would be useful to introduce a cortisol test measurement to such a study. This could help to understand to what extent the temporal short activation of autonomic adaptive mechanisms can be prolonged, after a short stress response period. It would enable to see if some students stay in a constant activated state during class activities or if some of class activities may induce a longer stress.

Finally, even if this study was not designed to see the interaction between stress events and performance on classroom assessments, to study the latter interaction is the most interesting perspective for educational research. Some previous literature suggests that the physical arousal which accompanies class tests and exams may increase attention (Zeidner and Matthews, 2005), improving focus and concentration on simple or repetitive tasks (Hodge et al., 1997; McDonald, 2001), or may have no impact at all (Wine, 1982; Chapell et al., 2005). However, other authors, like Anderson and Sauser (1995), argue that autonomic arousal is a necessary component of anxiety construct and therefore plays an important role. Moreover, Linnenbrink and Pintrich (2004) underline the fact that the difference in autonomic arousal can alter cognitive processing (consistent with the Yerkes-Dodson Law, 1908) and this relationship needs to be studied more. In this perspective, it would be enriching to study what is the relation between the periods of stress responses and cognitive performance during classroom activities compared to simple sympathetic arousal. These findings could broaden our understanding of the influence of stress response on cognitive (or sensomotor) performance, or at what level of stress, the performance can be impaired. It may also contribute to better understanding the degree of optimal vigilance necessary for the effective cognitive performance and enlarge the understanding of nature of the link between stress, affect, physiology, and learning.

## Data Availability Statement

Datasets are available on request. The raw data supporting the conclusions of this manuscript will be made available by the authors, without undue reservation, to any qualified researcher.

## Ethics Statement

This study was carried out in accordance with the recommendations of the guidelines for Ethical Research of the Ethical Research Committee of Saint-Petersburg State University with written informed consent from all subjects. All subjects gave written informed consent in accordance with the Declaration of Helsinki. The protocol was approved by the Ethical Research Committee of Saint-Petersburg State University.

## Author Contributions

VP is the main author of the article, who designed, carried out the study, and analyzed all data presented and also wrote theoretical part of the article as well as its experimental part. SK participated in the design of the study, provided the link between the university and the administration of the school where the study was carried out, and took part in registration, collection, and analysis of the data. SP enabled the experimental neurophysiological method of HRV analysis for the study and provided expertise on the collection and analysis of the stress data and provided a technical support. FF provided expertise and advice on data analysis as well as took part in carrying out the statistical analysis.

### Conflict of Interest

The authors declare that the research was conducted in the absence of any commercial or financial relationships that could be construed as a potential conflict of interest.
